# Genetic Analysis Based on CRISPR/Cas9 Technology in Plants

**DOI:** 10.3390/ijms242216398

**Published:** 2023-11-16

**Authors:** Kwon-Kyoo Kang, Yong-Gu Cho

**Affiliations:** 1 Division of Horticultural Biotechnology, Hankyong National University, Anseong 17579, Republic of Korea; 2 Department of Crop Science, Chungbuk National University, Cheongju 28644, Republic of Korea

Genome-editing technology is a type of genetic engineering in which DNA is inserted into, replaced in, or deleted from the genome using artificially engineered nucleases or genetic scissors. In other words, it is possible to create an altered DNA sequence through nuclease-mediated site-specific DNA cleavage via the DNA repair pathway. CRISPR/Cas9 is one of the most widely used gene-editing technology in plants, animals, and microorganisms because it is accurate, easy to use, convenient, and efficient among known genome editing related endonuclease. In early studies, the CRISPR/Cas9 system was programmed to perform RNA-guided DNA cleavage at specific sites in prokaryotes and has since proven to be an efficient tool for editing eukaryotic genomes [1]. Additionally, this system has also been applied in transcriptional regulation, epigenetic modification, and live-cell imaging via combination with other effector proteins [2]. The success of plant breeding is determined by phenotypic diversity and the genetic diversity of a population. Genome editing provides information for generating new alleles, fixing incorrect alleles, and pyramiding alleles to achieve a desired phenotype [3]. CRISPR/Cas9 technology has been widely used to improve agricultural traits related to yield, quality, disease resistance, nutrition, and domestication [4]. Indeed, an Editorial in the *International Journal of Molecular Sciences* (IJMS) about the development of new variants using CRISPR/Cas9 technology implies as much. Valuable information included in the current update concerns genes that play a role in ensuring increased yields and improved quality, abiotic stress tolerance, and biotic stress tolerance. However, in order to apply this information to plant breeding, genetic analysis of the corrected genes must be performed. This Special Issue of *IJMS* titled “Genetic analysis based on CRISPR/Cas9 technology in plants” includes six contributions. These six original articles are research articles documenting the editing of genes using CRISPR/Cas9 and the performance of genetic analysis in the T_2_ generation.

Song et al. [5] investigated 248 mutant plants targeting *α*-1,3-fucosyl transferase 1 (*FucT1*) and *β*-1,2-xylosyl transferase 1 (*XylT1*) genes using the CRISPR/Cas9 system and found that the mutation rates were 22.5% and 25%, respectively, and that both genes were 20.5% mutated. In the T_0_ generation, individuals showing hetero characteristics in the NbFucT1 locus were separated as chimeric offspring (37–54%), and individuals showing homo characteristics in the NbFucT1 locus produced many (~70%) homozygous mutation in the T_1_ generation. In the T_0_ generation, homozygous and bi-allelic mutations were stably inherited in the next generation, and the Cas9-free mutation, the null plant, was selected in the next generation. The Ta 161-1 line, +1 bp inserted in *NbFucT1* and −4 bp deleted in *NbXylT1*, produced T_2_ progenies that were homozygous in both loci and did not contain external genes containing Cas9. This result will contribute to a better understanding of the behavior of Cas9 used in targeted mutagenesis in *N. benthamiana* and serve as a useful reference for developing strategies designed to ensure the production of stable homozygous mutant plants. Also, Shin et al. [6] addressed the subject of Self-incompatibility (SI), stating that it is the most important system for preventing inbreeding in many flowering plants. Chinese cabbage, a major crop in Korea, exhibits self-incompatibility (SI). SI is controlled by type 2A serine/threonine protein phosphatases (*PP2As*). The *PP2A* gene consists of 36 kDa catalyst C subunits, 65 kDa regulatory A subunits, and various regulatory B subunits (50–70 kDa). Among them, the CRISPR/Cas9 system was used to generate knockout mutants using the PP2A 55kDa B regulated subunit (*PR55/B*) gene located on the A05 chromosome. Tentative gene-editing lines were selected via PCR analysis and Southern bolt analysis. Finally, seed formation was confirmed via flower pollination. The T_2_ generation yielded T-DNA-free self-compatible plants. Using the CRISPR/Cas9 technique, this study confirmed that the *PR55/B* gene, a PP2A 55 kDa B regulatory subunit, is closely related to the SI of Chinese cabbage. Kim et al. [7] investigated a method for targeted mutagenesis and golden SNP replacement of the *LcyE* gene using a CRISPR/Cas9 geminiviral replicon system in rice mutagenesis experiments performed on the Dongjin variety, achieving precise modification of the *LcyE* loci with an efficiency of up to 90%. This HDR experiment was the *LcyE* allele (*LcyE*-H523L) derived from another culture containing a golden SNP replacement. The phenotype of the homologous recombination (HR) mutant obtained through the geminiviral replicon-based template delivery system was tangerine-colored, and the frequency was 1.32% of the transformed calli. Shin et al. [8] performed functional analysis of the *AGL19* and *AGL24* genes related to bolting and flowering using the CRISPR/Cas9 system in Chinese cabbage. The tentative E0 AGL-edited lines were analyzed using NGS analysis. Two AGL19-edited lines and four AGL24-edited lines presented particularly late bolting compared to the inbred line ‘CT001’. In addition, the E1 AGL editing line without T-DNA was selected from the next generation via bud pollination of the selected editing lines. In conclusion, this research reported that late bolting was observed in the *AGL* gene editing lines generating by the CRISPR/Cas9 system, confirming that the AGAMAKY-like 19 (*AGL19*) and *AGL24* genes are associated with bolting and flowering in Chinese cabbage. Kim et al. [9] generated an *SGR1*-knockout (KO) null line via CRISPR/Cas9-mediated gene editing and conducted RNA sequencing and gas chromatography–tandem mass spectrometry analyses to identify the differentially expressed genes (DEGs). The *Solanum lycopersicum* SGR1 (*SlSGR1*) KO null lines presented a notably turbid brown coloration and significantly higher chlorophyll and carotenoid levels than those in the wild-type (WT) fruit. Differential gene expression analysis revealed 728 DEGs between the WT and the sgr#1-6 line, including 263 and 465 downregulated and upregulated genes, respectively, with a fold-change >2 and an adjusted *p*-value < 0.05. Most of these DEGs have functions related to photosynthesis, chloroplasts, and carotenoid biosynthesis. The strong changes in pigment and carotenoid content resulted in the accumulation of key primary metabolites, such as sucrose and its derivatives (fructose, galactinol, and raffinose), glycolytic intermediates (glucose, glucose-6-phosphate, and fructose-6-phosphate), and tricarboxylic acid cycle intermediates (malate and fumarate) in the leaves and fruit of the SGR-KO null lines. This research provided new evidence for the mechanisms underlying the roles of SGR1 as well as the molecular pathways involved in chlorophyll degradation and carotenoid biosynthesis. Kim et al. [10] generated KO lines of the *OsPUB7* gene using the CRISPR/Cas9 system. T_2_ seeds were harvested by selecting PUB7-GE plants that were T-DNA free in the T_1_ generation. Drought and salinity stress experiments using the PUB7-GE system revealed stress-resistant phenotypes. In addition, these lines exhibited lower ion leakage and higher proline content than the wild-type. It was suggested that the *OsPUB7* gene acted as a negative regulator of drought and salinity stress. Therefore, the *OsPUB7* gene with a U-box/ARM domain in rice can provide useful genetic resources for generating climate-change-resistant crops accounting for biological stress resistance such as drought and salinity.

The papers proposed in this Special Issue can be directly used for plant breeding by selecting a null line in the T_2_ generation after gene editing. In fact, papers by other authors often only report mutations in the PAM region caused by CRISPR/Cas9. However, this Special Issue focused more on the development of plant-breeding materials than other papers by reporting on the genetic analysis of the T_2_ generation. Academic and industrial studies on gene editing appear to have taken different paths depending on their respective areas of interest. We also believe that a gene-editing database should be established so that gene-editing data can be linked, and we contend that academia and industry should cooperate to develop gene-editing technology.
